# Selective Activation of D3 Dopamine Receptors Ameliorates DOI-Induced Head Twitching Accompanied by Changes in Corticostriatal Processing

**DOI:** 10.3390/ijms24119300

**Published:** 2023-05-26

**Authors:** Ana María Estrada-Sánchez, Claudia Rangel-Barajas, Andrew G. Howe, Scott J. Barton, Robert H. Mach, Robert R. Luedtke, George V. Rebec

**Affiliations:** 1 Program in Neuroscience and Department of Psychological and Brain Sciences, Indiana University, Bloomington, IN 47405, USA; crangelb@indiana.edu (C.R.-B.); sjbarton@iu.edu (S.J.B.); rebec@indiana.edu (G.V.R.); 2 División de Biología Molecular, Instituto Potosino de Investigación Científica y Tecnológica (IPICYT), Camino a la Presa San José No. 2055, Colonia Lomas 4a Sección, San Luis Potosi C.P. 78216, Mexico; 3 Psychology Department, University of California Los Angeles, Los Angeles, CA 90095, USA; ahowe@ucla.edu; 4 Intelligent Systems Laboratory, HRL Laboratories, LLC., Malibu, CA 90265, USA; 5 Department of Radiology, University of Pennsylvania School of Medicine, Chemistry Building, 231 S. 34th St., Philadelphia, PA 19104, USA; rmach@pennmedicine.upenn.edu; 6 Department of Pharmacology and Neuroscience, University of North Texas Health Science Center, 3500 Camp Bowie Boulevard, Fort Worth, TX 76107, USA; robert.luedtke@unthsc.edu

**Keywords:** 2,5-dimethoxy-4-iodoamphetamine (DOI), WC 44, WW-III-55, cortical pyramidal neurons, striatal medium spiny neurons, head-twitch response, mice

## Abstract

D3 receptors, a key component of the dopamine system, have emerged as a potential target of therapies to improve motor symptoms across neurodegenerative and neuropsychiatric conditions. In the present work, we evaluated the effect of D3 receptor activation on the involuntary head twitches induced by 2,5-dimethoxy-4-iodoamphetamine (DOI) at behavioral and electrophysiological levels. Mice received an intraperitoneal injection of either a full D3 agonist, WC 44 [4-(2-fluoroethyl)-N-[4-[4-(2-methoxyphenyl)piperazin 1-yl]butyl]benzamide] or a partial D3 agonist, WW-III-55 [N-(4-(4-(4-methoxyphenyl)piperazin-1-yl)butyl)-4-(thiophen-3-yl)benzamide] five minutes before the intraperitoneal administration of DOI. Compared to the control group, both D3 agonists delayed the onset of the DOI-induced head-twitch response and reduced the total number and frequency of the head twitches. Moreover, the simultaneous recording of neuronal activity in the motor cortex (M1) and dorsal striatum (DS) indicated that D3 activation led to slight changes in a single unit activity, mainly in DS, and increased its correlated firing in DS or between presumed cortical pyramidal neurons (CPNs) and striatal medium spiny neurons (MSNs). Our results confirm the role of D3 receptor activation in controlling DOI-induced involuntary movements and suggest that this effect involves, at least in part, an increase in correlated corticostriatal activity. A further understanding of the underlying mechanisms may provide a suitable target for treating neuropathologies in which involuntary movements occur.

## 1. Introduction

Pyramidal neurons in the primary motor cortex (M1) project to medium spiny neurons in the dorsal striatum (DS), forming the corticostriatal pathway that, among other functions, shapes and controls the motor output [1,2]. Altered corticostriatal processing is believed to underlie the development of the spontaneous, involuntary movements that are characteristic of neurodegenerative diseases such as Huntington’s and Parkinson’s and neuropsychiatric conditions such as Tourette’s syndrome [3,4]. Dopamine, a well-known modulator of corticostriatal processing, is often the target of therapies that are designed to improve these motor symptoms [5]. To date, however, the side effects of prolonged changes in dopamine transmission hamper the use of dopaminergic drugs in the clinic [6], keeping the search alive for improved dopaminergic therapies.

Based on their molecular structure and biological-induced responses, dopamine receptors can be divided into two main families: D1-like (including D1 and D5 receptors) and D2-like (including D2, D3 and D4) [7]. While the activation of the D1-like family activates adenylyl cyclase, which increases the activity of protein kinase A, D2 activation inhibits adenylyl cyclase [7]. The dopamine D3 receptor, a Gi-coupled receptor member of the D2-like dopamine family, is mainly located in the nucleus accumbens, the island of Calleja, and DS and has emerged as a potential therapeutic target [8,9,10]. The activation of the D3 receptor inhibits adenylyl cyclase and its second messenger pathway and has been linked to reward, motivation, and cognition, which may explain why D3 blockade has proven beneficial in animal models of drug abuse and schizophrenia (for review, see [11]). Interestingly, D3 receptors might also be involved in shaping motor behavior since they are expressed in the direct striatonigral pathway, which is implicated by the onset of movement [12], and the modulation of this pathway alleviates involuntary motor responses [13,14]. For example, the activation of D3 receptors by [4-(2-fluoroethyl)-N-[4-[4-(2-methoxyphenyl)piperazin 1-yl]butyl]benzamide] (WC 44), a full agonist, and 55 [N-(4-(4-(4-methoxyphenyl)piperazin-1-yl)butyl)-4-(thiophen-3-yl)benzamide] (WW-III-55), a partial agonist, reduces the involuntary, rapid movements of the head known in mice as the head-twitch response, which is induced by the 2,5-dimethoxy-4-iodoamphetamine (DOI; [15]). DOI, an agonist of the serotonin 5HT2A receptor, has been used to model Tourette’s-like behavior [16,17].

In the present study, we evaluated the protective effect of the D3 receptor activation on DOI-induced head twitching at a behavioral level and the underlying changes in neuronal processing behind them. We simultaneously recorded the activity of presumed cortical pyramidal neurons (CPNs) in M1 and medium spiny neurons (MSNs) in the DS of freely behaving mice injected with WC 44, WW-III-55, or a vehicle five minutes before DOI. Our results indicated that D3 receptor activation effectively reduced DOI-induced involuntary movements, which was partly underlined by changes in the corticostriatal activity.

## 2. Results

### 2.1. Dopamine D3 Receptor Agonists Reduce DOI-Induced Head Twitching

**Figure 1:**
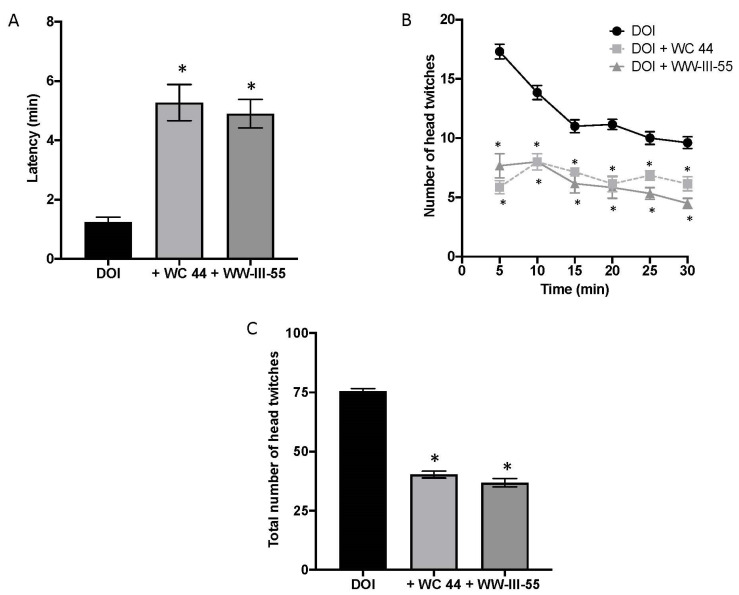
Activation of dopamine D3 receptors ameliorated DOI-induced head twitching. (**A**) DOI-induced head twitching started around 1.23 ± 0.175 min after its administration. In both cases, the injection of D3 receptor agonists (5 min before DOI), WC 44 and WW-III-55 increased the latency for the onset of head twitching (5.27 ± 0.61 for WC 44 and 4.89 ± 0.48 for WW-III-55 F (2, 17) = 24 (*p* < 0.0001). (**B**) Likewise, the temporal course of head twitching indicated that D3 agonists effectively reduced the presence of head twitching during the following 30 min (*p* < 0.005 relative to its corresponding time point). (**C**) As a consequence, D3-treated groups showed a significant reduction in the total number of head twitching events (40.29 ± 1.44 and 36.83 ± 1.75, WC 44 and WW-III-55, respectively), (F (2, 17) = 193.2 (*p* < 0.0001)). Control n = 7; WC 44 + DOI n = 7 and WW-III-55 + DOI n = 6. Statistically different from the control (*).

The onset of DOI-induced head twitching occurred within 1.23 ± 0.175 (n = 7) min in vehicle-pretreated mice but was significantly delayed by pretreatment with either D3 agonist, WC 44 (5.27 ± 0.61; n = 7) or WW-III-55 (4.89 ± 0.48; n = 6), F (2, 17) = 24.18 (*p* < 0.0001), as shown in Figure 1A. D3 receptor activation also significantly reduced the number of head twitches over the proceeding 30 min (Figure 1B) (*p* < 0.005). In fact, while DOI induced a total of 76 ± 1.54 head twitches (Figure 1C), WC 44 and WW-III-55 significantly decreased the total number of events (40.29 ± 1.44 and 36.83 ± 1.75, respectively) F(2, 17) = 193.2 (*p* < 0.0001). These results are comparable with the reduction in DOI-induced head twitching previously reported for D3 full agonists (WC 44) and partial agonists (WC 10, LAX-4-136 and WW-III-55), in DBA/2J mice [15].

### 2.2. Effect of D3 Receptor Agonists on DOI-Induced Changes in M1 and DS Spike Activity

**Figure 2:**
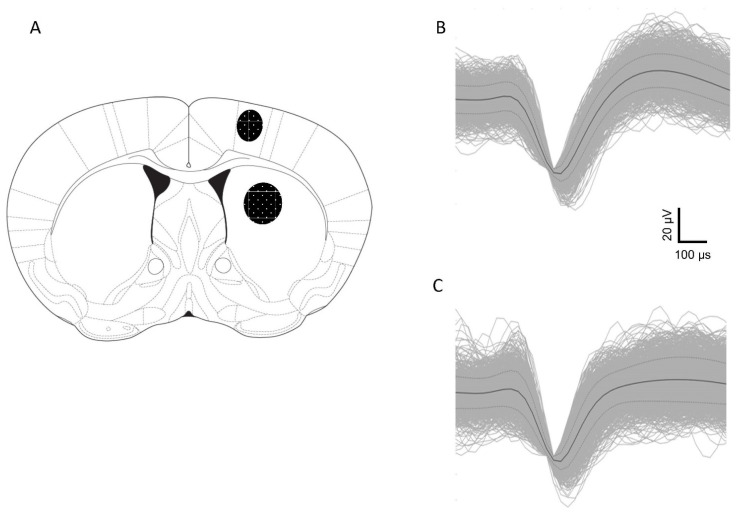
Schematic representation of electrode placements corroborated by histological analysis. (**A**) Shading indicates the location of placements in M1 cortex and DS as indicated at the coronal section +0.5 mm anterior to bregma. A representative waveform for a putative M1 pyramidal neuron (**B**) and a DS medium spiny neuron (**C**).

We evaluated changes in the activity of the presumed CPNs in M1 (Control n = 85; DOI n = 32; WC 44 + DOI n = 65; WW-III-55 + DOI n = 62 neurons) and MSNs in DS (Control n = 56; DOI n = 30; WC 44 + DOI n = 37; WW-III-55 + DOI n = 33 neurons). Electrode placements are shown schematically in Figure 2A, along with sample waveforms in M1 (Figure 2B) and DS (Figure 2C).

**Figure 3:**
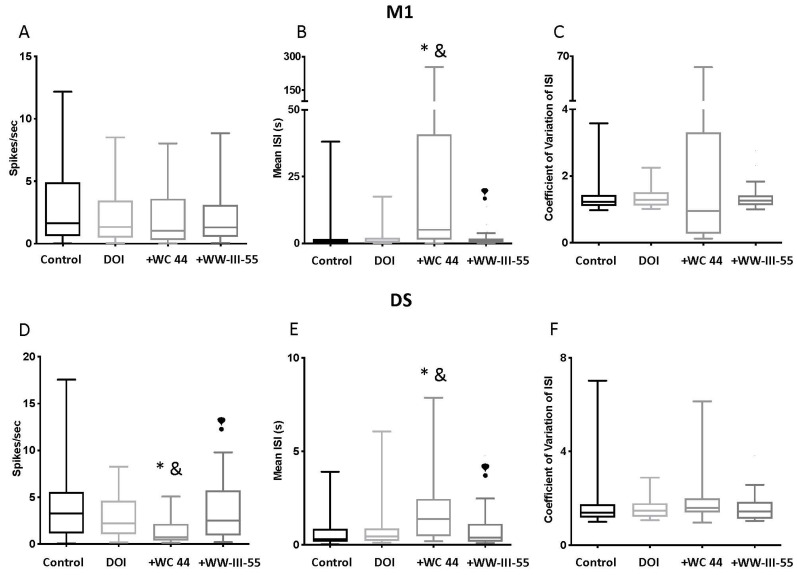
Spike activity in M1 and DS. The upper panels (**A**–**C**) show the electrophysiological spike activity recorded in M1. No changes in the firing rate and coefficient of variation in the inter-spike interval were observed in M1 (**A**,**C**); (*p* > 0.99). During WC 44 + DOI (shown in the graph as + WC 44), M1 neurons showed a significant increase in the mean inter-spike intervalrelative to the control (*p* < 0.0001) and DOI (*p* = 0.0001). A significant difference was observed (*p* < 0.0001) when comparing the mean inter-spike interval between the WC 44 + DOI and WW-III-55 + DOI (**B**). The lower panels (**D**–**F**) showed spike activity in DS. In DS, WC 44 caused a significant decrease in the firing rate relative to the control (*p* < 0.0001) and DOI (*p* = 0.0277) and was different from the effect caused by WW-III-55 + DOI (shown as +WW-III-55; *p* = 0.0021), while no changes were detected with WW-III-55 relative to the control or DOI (**D**). WC 44 + DOI showed a significant increase in the mean inter-spike interval compared to the control (*p* < 0.0001), DOI (*p* = 0.0247) and WW-III-55 + DOI (*p* = 0.0022) (**E**). Pretreatment with either WC 44 or WW-III-55 did not modify the CV ISI in DS (*p* > 0.3). Statistically different from the control (*), DOI (&), and WC 44 (❢).

Compared with the control group, the firing rate in M1 was unchanged by pretreatment with either the full or partial D3 agonist (*p* > 0.99; Figure 3A). Likewise, no significant changes were detected in the coefficient of variation in the inter-spike intervals (CV ISI) (*p* > 0.99) between groups. Only WC 44 + DOI showed a significant increase in the mean inter-spike interval relative to the control (*p* < 0.0001) and the DOI group (*p* = 0.0001), which is an indicator of spike-train variability (Figure 3B). Interestingly, when comparing the mean inter-spike interval between the WC 44 + DOI and WW-III-55 + DOI, a significant difference was observed (*p* < 0.0001). DS neurons, on the other hand, showed a significant decrease in the firing rate in the WC 44 + DOI group relative to the control (*p* < 0.0001) and DOI (*p* = 0.0277), which was also significantly different from the WW-III-55 + DOI group (*p* = 0.0021; Figure 3D). In line with decreased firing rates, WC 44 + DOI also showed a significant increase in the mean inter-spike interval compared to the control (*p* < 0.0001), DOI (*p* = 0.0247), and the WW-III-55 + DOI (*p* = 0.0022) groups (Figure 3E). As in M1, pretreatment with either WC 44 or WW-III-55 did not modify the CV ISI (*p* > 0.3; Figure 3F).

**Figure 4:**
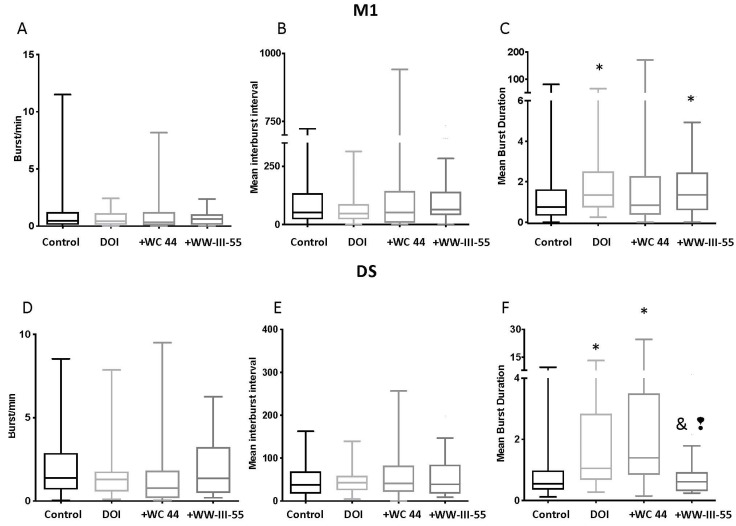
Burst activity in M1 and DS. M1 neurons showed no change in their burst rate, mean inter-burst interval, and mean burst duration by WC 44 + DOI and WW-III-55 + DOI compared to DOI (*p* > 0.09) (**A**–**C**). DOI and WW-III-55 + DOI significantly increased the mean burst duration relative to the control (*p* = 0.008 for DOI and *p* = 0.010 for WW-III-55 + DOI; (**C**). In DS, the mean burst rate and mean inter-burst interval were unchanged (**D**,**E**). In the WW-III-55 + DOI group, DS neurons showed a significant decrease in the mean burst duration compared to DOI (*p* = 0.0042) and WC 44 + DOI (*p* < 0.0001; (**F**). Both DOI and + WC 44 + DOI showed a significant increase in the mean burst duration compared to the control (*p* = 0.0042 for DOI and *p* < 0.0001 for WC 44 + DOI). Statistically different from the control (*), DOI (&), and WC 44 (❢).

The DOI-induced burst rate, mean inter-burst interval and mean burst duration was unchanged by WC 44 and WW-III-55 (*p* > 0.23; Figure 4A–C) in M1. Only DOI (*p* = 0.008) and WW-III-55 + DOI (*p* = 0.010) showed a significant increase in the mean burst duration relative to the control (Figure 4C). Similarly, in DS, the mean burst rate and mean inter-burst interval were unchanged (Figure 4D,E). However, DS neurons in DOI (*p* = 0.0042) and WC 44 + DOI (*p* < 0.0001) showed a significant increase in the mean burst duration relative to the control group. The WW-III-55 + DOI group showed a significant decrease in their mean burst duration compared to DOI (*p* = 0.0125) and WC 44 + DOI (*p* = 0.0005; Figure 4F).

Collectively, these results indicated that the activation of D3 receptors followed by the administration of DOI had a greater effect on firing patterns in DS than in M1. Interestingly, however, the changes in DOI spike activity induced by WC 44 and WW-III-55 were different, suggesting that a partial versus a full D3 agonist might contribute to the neuronal activity changes elicited by the degree of activation in D3 receptors.

**Figure 5:**
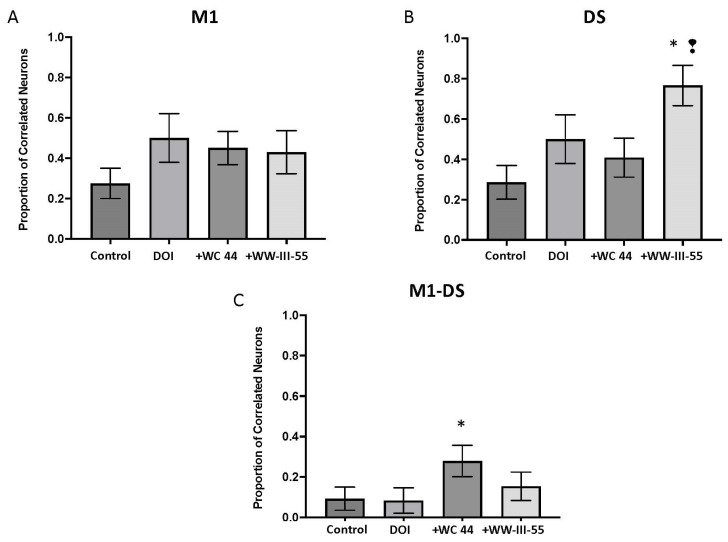
Correlated activity between M1 and DS. The proportion of correlated activity between pairs of neurons simultaneously recorded in M1 (**A**) and DS (**B**) and between M1 and DS (**C**) was evaluated by the number of correlated pairs divided by the total number of pairs. The WW-III-55 + DOI group showed a significant proportion of correlated neurons relative to the control (*p* = 0.0008), while WC 44 showed a significant increase in correlated pairs between M1 and DS (*p* = 0.02). Statistically different from the control (*) and WC 44 (❢).

To evaluate the correlated activity between pairs of simultaneously recorded neurons, we constructed cross-correlation histograms and calculated the proportion of correlated pairs. In M1 and DS, DOI increased the proportion of correlated pairs that were relative to the control, although this effect was not statistically significant (*p* = 0.08, *p* = 0.10, respectively; Figure 5A,B). For both brain structures, WC 44 and WW-III-55 failed to change DOI-induced correlated activity (M1 *p* = 0.77, DS *p* = 0.77 for WC 44; M1 *p* = 0.51, DS *p* = 0.11 for WW-III-55; Figure 5A,B). In DS, only WW-III-55 significantly increased the proportion of correlated neurons relative to the control (*p* = 0.0008) and WC 44 (*p* = 0.01), Figure 5B. The proportion of correlated neurons between M1 and DS was significantly increased only in the WC 44 group relative to the control (*p* = 0.02; Figure 5C).

## 3. Discussion

The activation of serotonin 5HT2A receptors by DOI led to an involuntary head twitch response in mice, which occurred along with dysregulated corticostriatal activity [18]. Here, we found that pretreatment with a full (WC 44) or partial (WW-III-55) D3 agonist delayed the onset and reduced the frequency and the total number of DOI-induced head twitches. Furthermore, our electrophysiological data showed that D3 activation with both agonists had no effect on the CPN firing rate or burst activity, which was consistent with the low expression of D3 receptors in cortical neurons [19]. However, an increase in the CPN and mean ISI could reflect the action on extra-cortical receptors (i.e., activation of interneurons) likely due to systemic injection of WC 44. WW-III-55 + DOI, by contrast, had no effect on any parameter of our CPN spike analysis. In the striatum, pretreatment with WC 44 led to a reduced MSN firing rate and an increase in the mean ISI relative to DOI, while WW-III-55 only reduced the mean duration of the MSN burst. CPN and MSN spike analysis showed that the full agonist WC 44 mainly changed DS firing activity, with a lesser effect on WW-III-55 pretreated mice, suggesting that the main effect of D3 activation was exerted in DS: a brain area with the preferential expression of D3 receptors [19]. These results were consistent with the pharmacological characterization of these compounds: WC 44 reduced 96% of the adenylyl cyclase intracellular responses, whereas WW-II-55 inhibited 67% of the adenylyl cyclase activity [15].

The identified electrophysiological changes indicated that DS, rather than M1, was the likely source of the output signal triggered by the activation effect of D3 receptors. A response to M1 was also expected, although to a lesser degree since both structures constitute the brain motor circuit where M1 projected to the striatum, which sent the information through direct and indirect pathways to the thalamus, and this structure projected back up to the cortex [20]. Therefore, to better understand the effects of D3 activation on this motor circuit and how it reduced the presence of involuntary movements, electrophysiological analysis of the thalamus could increase our understanding of neuronal information flow downstream of the striatum.

The evaluation of correlated firing between pairs of simultaneously recorded cortical and striatal neurons failed to reveal more information on the corticostriatal changes elicited by the activation of the D3 receptors and the improved DOI-induced head twitching. However, it was interesting that relative to the control group, an increased correlated activity was detected between the DS pair of neurons after WW-III-55 + DOI treatment, suggesting that a more correlated activity in DS neurons could occur after D3 activation. An effect that was not observed with WC 44 + DOI in the DS, but was present in the correlated firing between M1 and DS. As WC 44 was a full agonist, the effect of correlated activity might spread across the motor brain control circuitry, including the basal ganglia components and the thalamus, which might lead to an increased correlated activity between M1 and DS. Furthermore, simultaneous recordings of the local field potential of these structures could provide more information on the connectivity between M1 and DS during the activation of D3 receptors.

Changes in the correlated activity between neurons are a key factor in animal models that developed involuntary movements. According to a previous report, DOI-induced head twitching increased the correlated activity in DS and between M1 and DS [18]. On the contrary, transgenic models of Huntington’s disease, a CAG-related neurodegenerative disorder characterized by the progressive development of involuntary movements, show reduced correlated activity between the striatal neurons [21]. Although M1 showed slight changes in electrophysiological properties in this study, the evaluation of Huntington’s disease models also indicated that changes in the correlated neuronal activity in M1 also contributed to impaired corticostriatal neuronal functioning and the progressive appearance of the motor phenotype [22]. Additionally, of note was the evidence that abnormal neuronal synchronized activity has been reported in other neurodegenerative conditions, such as Parkinson’s and Alzheimer’s disease and other neuropathologies such as Tourettes and autism (For review, see [23]).

Moreover, our results suggested that when the activation of D3 receptors preceded the development of involuntary movements, corticostriatal circuitry could cope with the changes induced by DOI that lead to head twitching. An interesting question emerges if the activation of the D3 receptors after the presence of involuntary movements could have the same protective effect. This question was relevant for the potential use of D3 agonists in brain disorders characterized by involuntary movements: a factor that needs to be evaluated in future studies. Nonetheless, our results support that the activation of D3 receptors might be a therapeutic target to alleviate involuntary movements in neurodegenerative conditions such as Tourette’s, Huntington’s, and Parkinson’s diseases or the involuntary motor side effect of already used treatments. For example, levodopa, a drug used to manage Parkinson’s disease, unfortunately, also leads to dyskinesia. Interestingly, the activation of D3 receptors effectively reduced levodopa-induced dyskinesia [24].

The striatum was central to the motor brain control circuitry. As its alteration could progressively lead to involuntary movements, or as described in the present study, D3 activation in the striatum might also reduce the presence of involuntary movements. Therefore, the integrity of the striatum circuitry might be a factor to consider during the evaluation of the D3 agonist as a possible target.

Our analysis mainly focused on the contribution of cortical and striatal neurons. However, D3 receptors are also expressed in striatal acetylcholine interneurons [25] and at presynaptic sites in dopaminergic terminals [26]. Moreover, growing evidence supports the role of astrocytes in actively modulating the synapses and behavioral output (For review, see [27,28]). In this regard, basal ganglia astrocytes express D3 receptors [29]. Therefore, evaluating the contribution of interneurons and astrocytes during the activation of D3 receptors might provide more information about the corticostriatal changes that underlie a reduction in the DOI-induced head-twitching response.

One limitation of our study is that we only evaluated the effects of WC 44 and WW-III-55 for the first thirty minutes after they were administered. While we observed a clear reduction in DOI-induced head twitches, evaluating the effects of D3 activation beyond this period could be important to understand the long-term effect on behavior and electrophysiology changes. Additionally, studying different doses of WC 44 and WW-III-5 could help us better understand their potential therapeutic applications.

The present study demonstrated that the partial or full activation of D3 receptors could alleviate the involuntary motor responses induced by DOI that occurred along with changes in corticostriatal neuronal activity, such as increased correlated activity in DS. These data support the potential role of D3 receptors as a therapeutic target for brain diseases in relation to the development of involuntary movements.

## 4. Materials and Methods

### 4.1. Animal Care and Housing

Animal use was in accordance with the National Institutes of Health Guide for the Care and Use of Laboratory Animals and was approved by the local Institutional Animal Care and Use Committee. All efforts were made to minimize suffering and the number of animals used in these experiments. FvB/N background male mice (n = 22) from 4 to 6 months old were housed in the Psychology building animal facility at Indiana University, Bloomington, and were maintained under controlled temperature and humidity conditions with a 12 h light/dark cycle with free access to food and water.

### 4.2. Electrodes and Implantation Surgery

Electrode bundles were built in-house. As described elsewhere [30], each bundle consisted of 4 recording electrodes (25 μm diameter insulated stainless steel micro-wires) and 50 μm diameter uninsulated stainless-steel ground wire (California Fine Wire, Grover Beach, CA, USA). Each wire was friction-fitted to gold-plated pin connectors into polyphenylene sulfide insulators (7 × 6 × 4 mm) (Omnetics Connector Corporation, Minneapolis, MN, USA). To record M1 and DS simultaneously, 2 bundles were glued together, and one was cut to 0.5 mm for M1 and the other to 3.0 mm for DS.

For electrode implantation, after a subcutaneous meloxicam injection (1 mg/kg), mice were anesthetized with a mixture of chloral hydrate and sodium pentobarbital (chloropent: 170 mg/kg chloral hydrate and 40 mg/kg sodium pentobarbital), which was administered at 0.4 mL/100g body weight (intraperitoneally), and the mice were mounted in a stereotaxic frame. After the skull was exposed, a hole was drilled +0.5 mm into the anterior and ±1.5 mm lateral to the bregma [31]. Two additional holes were drilled in the contralateral hemisphere for the placement of stainless-steel anchor screws. After the electrode bundles were lowered into M1 and DS (0.5 mm and 3.0 mm ventral to the brain surface, respectively), the electrode assembly and anchor screws were fixed in place with dental acrylic. Mice were allowed to recover fully, and recordings began one week after implantation surgery.

### 4.3. Drugs and Experimental Protocol

DOI (Sigma Aldrich Research Biochemicals Inc., Burlington, MA, USA) was administered intraperitoneally at a dose (5 mg/kg) that was known to induce head twitching in FvB/N mice [18]. To study the effects of the activation of D3 receptors on DOI-induced corticostriatal changes and head twitching, we tested a full agonist 4-(2-fluoroethyl)-N-[4-[4-(2-methoxyphenyl)piperazin 1-yl]butyl]benzamide (WC 44) and a partial agonist N-(4-(4-(4-methoxyphenyl)piperazin-1-yl)butyl)-4-(thiophen-3-yl)benzamide (WW-III-55); these compounds were developed by [32,33]. We used a dose of 5 mg/kg WC 44 and 6 mg/kg WW-III-55. These doses were chosen based on dose–response curves, using the IC50 value, which effectively reduced the number of DOI-induced head-twitch events [15].

Mice received an intraperitoneal injection of either WC 44, WW-III-55, or a vehicle five minutes before the administration of DOI. The mice were allowed to behave in an open-field arena where neuronal activity and behavior were recorded simultaneously for a total of 35 min (5 min after the first injection, and 30 min after DOI). Neuronal activity in M1 and DS was recorded for 30 min and a different group was used as a control. Two independent (blind) observers identified instances of head twitching, which was defined as a rapid movement of the head left to right or vice versa (without the involvement of the front paws), and quantified the number of head twitches in 5 min intervals.

### 4.4. Behavioral Electrophysiology

Neuronal activity was recorded during the light phase of the diurnal cycle while the mice freely explored the open-field arena located in a soundproof and electrically shielded chamber. Electrophysiological recordings were performed as previously described [34]. In brief, male gold pins attached to a lightweight, flexible wire harness and equipped with field-effect transistors were inserted into the head-mounted electrode assembly. A multichannel acquisition processor acquired neuronal discharges (Plexon, Dallas, TX, USA). Electrode signals were band-pass filtered (154 Hz to 8.8 KHz) and digitized at a rate of 40 KHz.

All spike sorting occurred online before the beginning of the drug administration and the recording session. Sort Client software (Plexon, Dallas, TX, USA) was used in conjunction with oscilloscope tracking to isolate each unit (matching the analog signal with the digitized template) and to eliminate the need for post hoc offline sorting. A voltage threshold of 2.5-fold background noise was established, and a template waveform was created via component analysis. Recorded units were treated as independent entities in each recording session because electrode drift and subtle changes in the behavioral state could not guarantee the positive detection of the same neuron over multiple sessions [35]. Presumed interneurons, characterized by a high-frequency firing rate (≥10 spikes/s), were excluded.

Following the isolation and identification of units, we followed the drug administration protocol (see above).

### 4.5. Spike-Train Analysis

Electrophysiological data were analyzed with NeuroExplorer (Nex Technologies, Littleton, MA) and MATLAB (Mathworks, Natick, MA, USA) scripts. The firing rate was calculated by dividing the spike trains into 1-s bins (spikes/s). Spike-train variability was evaluated through the coefficient of variation in interspike intervals (CV ISIs), which were calculated by dividing the standard deviation of all ISIs in a train by the mean ISI of the train. The Poisson surprise method was used to calculate bursting activity with brief periods of high-frequency firing [36,37]. This method used a probability-based approach to burst detection that determined if a set of consecutive ISIs occurred with a sufficiently low probability; the event was considered “surprising” and classified as a burst. The surprise value indicated how intense or “surprising” the ISIs of a particular burst were in relation to other ISIs in the same train and provided an estimate of the statistical significance of each burst in the train [30]. The following bursting properties were analyzed for each recorded neuron: burst rate, percent of spikes that occurred in bursts, mean burst surprise, mean burst duration, mean ISI in a burst, mean burst frequency, and mean number of spikes per burst.

To evaluate the correlated activity between pairs of neurons, cross-correlation histograms were constructed for each pairwise comparison in M1 and DS and between both structures for each recording session. The cross-correlation histograms were based on 0.5 ms bins and a 0.5-s time lag from the zero bin, which were smoothed using a Gaussian filter with a bin width of 3. Synchronized firing between the two simultaneously recorded neurons was identified by 99% confidence intervals (for more details, see [34]). We calculated the proportions of correlated neuronal pairs obtained within and between M1 and DS.

### 4.6. Histology

Electrode placements were verified after the completion of the final recording session. Mice were deeply anesthetized with double the surgical doses of chloropent, and each active microwire was marked with a current pulse (30 μA for 10 s). Mice were transcardially perfused with 0.9% saline solution followed by 10% paraformaldehyde. The brain was immediately removed and placed in a 10% paraformaldehyde solution containing 10% potassium ferrocyanide [K4Fe(CN)6] to produce small blue deposits at the site of the recording. A consecutive series of 40 μm coronal sections were obtained to verify the electrode placements in both M1 and DS.

### 4.7. Statistical Analysis

The statistical program package: GraphPad Prism version 9.5.1 (GraphPad Software, La Jolla, CA, USA) was used to analyze both electrophysiological and behavioral data. The onset of head twitching and the total number of head twitches were analyzed by one-way ANOVA, followed by Dunnett’s multiple comparisons test. The temporal response to a D3 agonist on head twitching was analyzed by two-way repeated measures of ANOVA followed by Tukey’s multiple comparisons tests. For spike data, which significantly deviated from normality and lacked the homogeneity of variance in spike-train samples, nonparametric statistics were used. Thus, we used the Kruskal–Wallis test followed by Dunn’s multiple comparison tests to assess the firing rate, CV ISI, and all burst parameters. Electrophysiological data were presented as box-and-whiskers plots; the box extended from the 25th to the 75th percentile with the line at the median (50th percentile), and the whiskers represented the minimum and maximum values. A Kruskal–Wallis test was followed by an uncorrected Dunn’s test, which was used to analyze the difference between the proportion of correlated neurons between the groups. Data were expressed as the mean ± SEM; differences were considered significant when *p* ≤ 0.05.

## 5. Conclusions

Pretreatment with the dopamine 3 (D3) receptor agonists WC 44 and WW-III-55 delayed the DOI-induced involuntary head twitch onset, number, and frequency. Simultaneous electrophysiological recordings of M1 and DS showed that D3 activation led to slight changes in the single unit activity, mainly in DS, and increased correlated firing in DS or between presumed CPNs and MSNs. These data suggest that D3 receptor activation could lead to changes in DS, which might underlie the reduced head twitch response elicited by DOI. Although these results support the fact that D3 receptor activation might be a suitable target for neuropathologies where involuntary movements occur, more studies are needed to evaluate the long-term effect of D3 receptor activation with WC 44 and WW-III-55.

## Data Availability

Data are available upon request.

## Abbreviations

DOI	2,5-dimethoxy-4-iodoamphetamine
DS	Dorsal striatum
CV ISI	Coefficient of variation in inter-spike intervals
M1	cortical motor area 1
WC 44	4-(2-fluoroethyl)-N-[4-[4-(2-methoxyphenyl)piperazin 1-yl]butyl]benzamide
WW-III-55	N-(4-(4-(4-methoxyphenyl)piperazin-1-yl)butyl)-4-(thiophen-3 yl)benzamide
